# Predictive Factors of Hospitalization and Emergency Visits Among Children With Asthma

**DOI:** 10.7759/cureus.51487

**Published:** 2024-01-01

**Authors:** Hussain A Al Ghadeer, Jalal K Aldandan, Marwah A Alessa, Sirar A Al Ali, Abdullah M Alajalin, Ali A Al Ghadeer, Hassan M Albahrani, Qasem I Alherz, Latifah A Almulhim, Ibrahim A Altaweel, Badah A Alqahtani, Ghadeer A Al Bensaad, Muntaha N Alnasser, Rawan H Alhumaid, Reham m Fatani

**Affiliations:** 1 Pediatrics, Maternity and Children Hospital, Al-Ahsa, SAU; 2 Pediatrics, New Medical Center (NMC) Royal Hospital, Sharjah, ARE; 3 Pediatrics, Prince Saud Bin Jalawi Hospital, Al-Ahsa, SAU; 4 Dentistry, Vision College, Riyadh, SAU; 5 Pediatrics, King Faisal University, Al-Ahsa, SAU; 6 Pediatrics, Maternity and Children Hospital, Jeddah, SAU

**Keywords:** saudi arabia, children, asthma control test, exacerbation, asthma

## Abstract

Introduction

A chronic diverse inflammatory disease, asthma affects millions of people worldwide. To control asthma, standardized care is essential. Children with asthma who receive appropriate care have lower emergency room (ER) visits and hospital stays as well as a higher quality of life than children who do not receive appropriate care. We aim to evaluate the predictive variables of hospitalization and ER visits in children with asthma.

Methodology

In 2022 and 2023, a cross-sectional descriptive study was carried out on children with asthma and their caregivers who were attending primary health care clinics in the eastern region of Saudi Arabia. We used the Childhood Asthma Control Test (C-ACT) to evaluate asthma control. A C-ACT score of less than 19 indicates uncontrolled childhood asthma. To investigate the relationships between the risk factors and the rate of ER visits and hospitalizations, we performed a multiple logistic descriptive analysis.

Results

In this study, 124 asthmatic children from primary health care centers matched the inclusion criteria. The majority of children had atopy, and their mean age was 10.8±3.4 years. Concerning the risk factors linked to ER visits and hospitalization, there is evidence that not following up with physicians, using more frequent and short-acting beta-agonists, exposure to smoke and household pets, and poor asthma control are linked to increased rates of both ER visits and hospitalizations.

Conclusion

Better asthma control in children and adolescents may be achieved by providing inexpensive asthma care services, more thorough parental and child education, and effective symptom management. These measures can help reduce exacerbations of asthma and the consequences that accompany them.

## Introduction

An estimated one-third of the world’s population suffers from allergic diseases. The most frequent allergy in the world, accounting for 400 million cases, is rhinitis, followed by 339 million asthma cases and 250 million people with food allergies. Because of alterations in the environment and the immune system, the prevalence of respiratory allergies has increased over the past decades [1,2]. The complex combinations of genetics, environmental exposure, and host variables lead to asthma, which is a chronic heterogeneous illness. Aeroallergens, the weather, and pollen are examples of environmental exposure. The host variables include dietary deficiencies, infections, allergic hypersensitivity, and obesity [3]. Airflow restriction is often what defines asthma. Respiratory symptoms, such as coughing, wheezing, shortness of breath, and chestiness, usually appear with varying degrees of intensity and severity. These symptoms vary with the time of day, being more common at night and in the early morning. They also get worse during specific seasons, particularly in the spring and fall when there is a rise in the triggering pollination [4,5]. Asthma is a major noncommunicable disease that has a considerable influence on both children’s and adults’ well-being and quality of life. Asthma affects around 339 million people worldwide, with an additional 100 million anticipated by 2025. Asthma is rated 16th and 28th in terms of years lived with disability and disease burden as measured by disability-adjusted life years [6,7].

According to the Centers for Disease Control and Prevention, the total prevalence of asthma in the United States in 2019 was 25.1 million persons, with 5.1 million (7%) of them being children, meaning that one out of every 14 children had asthma. About 2.3 million of these children had at least one asthma attack [8,9]. 

An acute or subacute worsening of asthma symptoms that necessitates medical attention is known as an exacerbation. An asthma attack may manifest as the initial sign of asthma, particularly in young patients. Viral infection is the most common cause of asthma exacerbations. Additional causes include intense motion, exercise, cold air, aspirin, and nonsteroidal anti-inflammatory medicines. Exacerbation of asthma has a substantial impact on the patient, their family, and society because it is related to missed school days and a low quality of life. Asthma was responsible for approximately 13.8 million absences on school days. Exacerbations of asthma can result in roughly 1.6 million visits to the emergency room (ER) [10]. Patients with moderate to severe asthma exacerbations have a greater rate of hospitalization [11]. In the United States, the expected number of hospitalizations due to asthma exacerbation was 183,000 [12]. Thus, in this study, we seek to predict the risk factors that are associated with rates of hospitalization and ER visits for children with asthma in the eastern region of Saudi Arabia.

## Materials and methods

This was a descriptive, cross-sectional, and analytical clinical study carried out at primary health care centers with their caregivers in 2022 and 2023 in the eastern region of Saudi Arabia. We included all children and adolescents diagnosed with asthma. To assess whether their asthma was under control, children and adolescents between 5 and 17 years old who had received an asthma diagnosis from a pediatrician at least six months prior to inclusion made up the study cohort. Clinically, asthma exacerbation is identified by the increasing worsening of dyspnea, coughing, wheezing, or tightness in the chest. The questionnaire was divided into three sections: sociodemographic and clinical characteristics of asthma, pharmacological asthma treatment, and an asthma control test.

Data collection

We used the Arabic version of the asthma control test recommended by the Saudi Initiative for Asthma in our data collection. A patient’s activity limits, dyspnea, frequency of nighttime symptoms, usage of rescue medication, and overall disease control rating over the last four weeks are covered by the five elements in this assessment. Responses to each question are scored on a scale of one (poor control) to five (excellent control). The total score range is five to 25; scores ≤ 19 are considered uncontrolled asthma, scores 20-24 are considered partially controlled, and scores of 25 are considered controlled asthma [13,14].

Data analysis

We collected, reviewed, and then fed the data into the Statistical Package for Social Sciences version 21 (SPSS, IBM Corp., Armonk, NY) ). All statistical methods used were two-tailed with an alpha level of 0.05 considering significance when the P-value was less than or equal to 0.05. We obtained the total asthma control test score by summing up all items’ discrete scores and then categorizing them into poor, partial, and well-controlled asthma categories using prescribed tool cutoff points [15]. We performed descriptive analysis by prescribing frequency distribution and percentage for study variables including children’s personal data, medical history, pattern and severity, and asthma risk factors. We also tabulated clinical management and medications and graphed triggers of asthma and asthma control levels. We cross-tabulated to show risk factors of ER visits and hospital admission using the Pearson chi-square test for significance and exact probability test if there were small frequency distributions.

## Results

There were a total of 124 eligible asthmatic children (67 females (54%)) 1-17 years with a mean age of 10.8±3.4 years. Regarding other atopy conditions, 43 (34.7%) had nasal allergies, 31 (25%) had eczema, 15 (12.1%) had nasal polyps, and eight (6.5%) had other health conditions (Table 1).

**Table 1:** Bio-demographic data study of children with asthma

Bio-demographic data	No	%
Age in years		
1-5	30	24.2%
6-11	39	31.5%
12-17	55	44.4%
Gender		
Male	57	46.0%
Female	67	54.0%
History of atopy		
Nasal allergy	43	34.7%
Eczema	31	25.0%
Nasal polyps	15	12.1%
Other comorbidities	8	6.5%
None	55	44.4%

Table 2 shows the pattern and severity of asthma among participating children in Saudi Arabia. Most caregivers did not know the severity of the child’s asthma; only 11.3% had a severity score of four or five. Also, 61 (49.2%) investigated for aeroallergens revealed that 43 (70.5%) were sensitive to dust, but not four (6.6%). Regarding risk factors, 58.9% had a family history of asthma, 33.9% had smokers at home, and 25% had a pet in the house. Also, 48 (38.7%) were exposed to triggers frequently. As for asthma pattern and effect, 25.4% experienced five or more asthmatic exacerbations per year, 23.4% visited the ER five times or more per year, 9.7% reported exercise-induced asthma exacerbation five times or more per year, and 6.4% had been hospitalized five times or more per year. Twenty-one (16.9%) had needed ICU admission due to asthma, and 10 (8.1%) had needed mechanical ventilation.

**Table 2:** Pattern, severity, and risk factors of asthma among study children

Pattern and severity		No	%
Severity of asthma	Not known	84	67.7%
	1	7	5.6%
	2	7	5.6%
	3	12	9.7%
	4	8	6.5%
	5	6	4.8%
Has your child been investigated for aeroallergens?	Yes	61	49.2%
	No	63	50.8%
If yes, what was the result?	Negative	4	6.6%
	Dust	43	70.5%
	Pollens	1	1.6%
	Animals	2	3.3%
	Others	11	18.0%
Family history of asthma	Yes	73	58.9%
	No	51	41.1%
Home smoker	Yes	42	33.9%
	No	82	66.1%
Is there a pet in the house?	Yes	31	25.0%
	No	93	75.0%
Is your child exposed to triggers frequently?	Yes	48	38.7%
	No	76	61.3%
Average asthmatic exacerbation (yearly)	Never	27	21.8%
	1-2 times	41	33.1%
	3-4 times	27	21.8%
	5-6 times	11	8.9%
	>6 times	18	14.5%
Average exercise induces asthma exacerbation (yearly)	Never	63	50.8%
	1-2 times	33	26.6%
	3-4 times	16	12.9%
	5-6 times	4	3.2%
	>6 times	8	6.5%
Average visit to the emergency department (yearly)	Never	42	33.9%
	1-2 times	38	30.6%
	3-4 times	15	12.1%
	5-6 times	16	12.9%
	>6 times	13	10.5%
Average hospitalization (yearly)	Never	84	67.7%
	1-2 times	21	16.9%
	3-4 times	11	8.9%
	5-6 times	2	1.6%
	>6 times	6	4.8%
Intensive care admission during the last year	Yes	21	16.9%
	No	103	83.1%
Have you had an asthma attack that required mechanical ventilation?	Yes	10	8.1%
	No	114	91.9%

**Figure 1:**
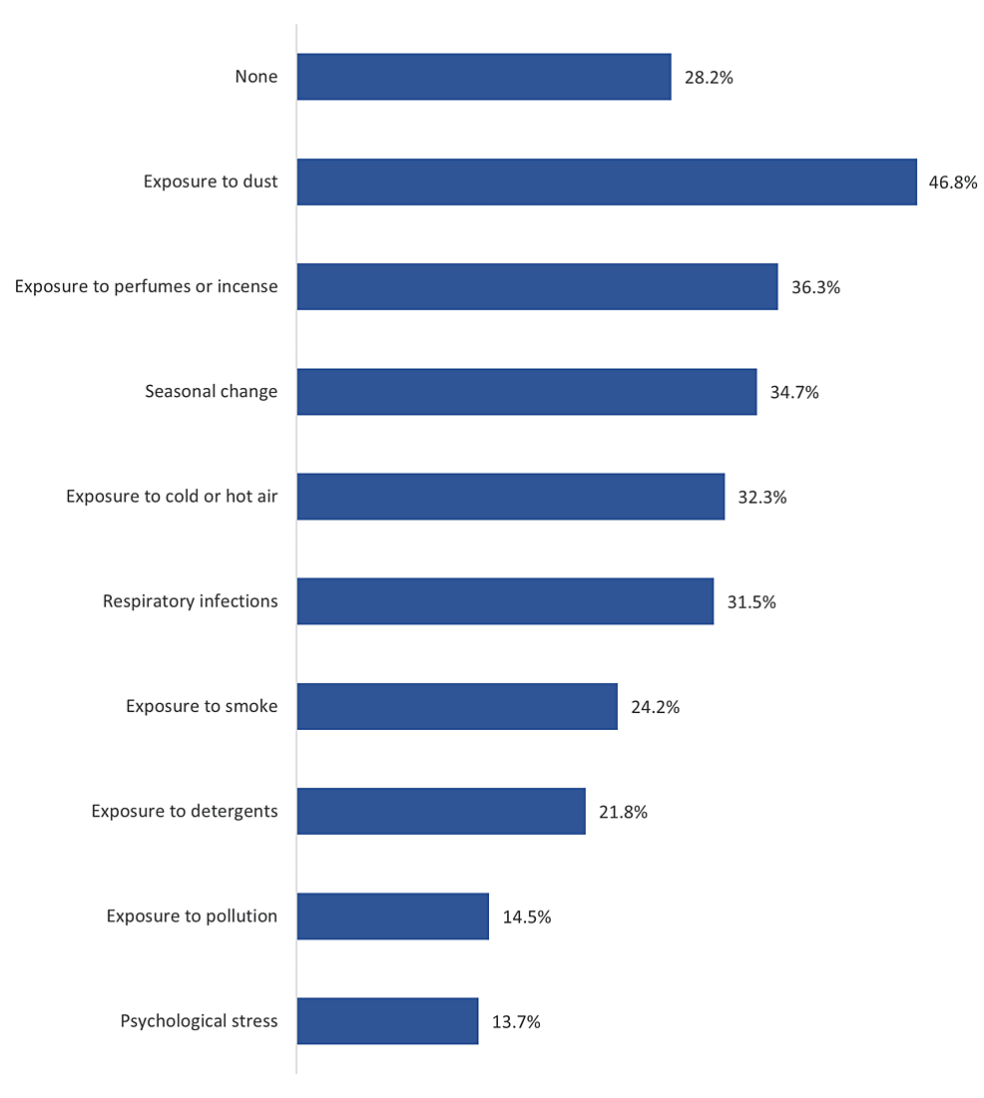
Triggers of asthma among study children

Figure 1 shows triggers of asthma among participating children, Saudi Arabia. The most reported triggers included exposure to dust (46.8%), exposure to perfumes or incense (36.3%), seasonal change (34.7%), exposure to cold or hot air (32.3%), respiratory infections (31.5%), and exposure to smoke (24.2%).

Table 3 shows clinical management and medications among participating children in Saudi Arabia. Fifty-six (45.2%) were on an inhaled corticosteroid, 20 (16.1%) were on a long-acting beta-agonist, 11 (8.9%) were on montelukast, and 10 (8.1%) were on inhaled steroids. Forty-one (33.1%) did not use prophylactic medication. Fifty-three (42.7%) did regular follow-ups with a pediatrician at the clinic. Most of the children (64.5%) reported that their physician checked the proper usage of their nebulizer or spacer.

**Table 3:** Clinical management and medications among asthmatic children

Management	No	%
On prophylactic medication		
None	41	33.1%
Inhaled corticosteroid	56	45.2%
Long-acting beta-agonist	20	16.1%
Montelukast	11	8.9%
Systemic steroids	10	8.1%
On regular follow-up with the pediatric clinic		
No	53	42.7%
Yes	71	57.3%
Does your physician check the proper usage of a nebulizer or spacer		
No	80	64.5%
Yes	44	35.5%
Usage of short-acting beta-agonist (monthly)		
Never	25	20.2%
1-2 times	42	33.9%
3-4 times	25	20.2%
5-6 times	12	9.7%
More than one bottle	20	16.1%

**Figure 2:**
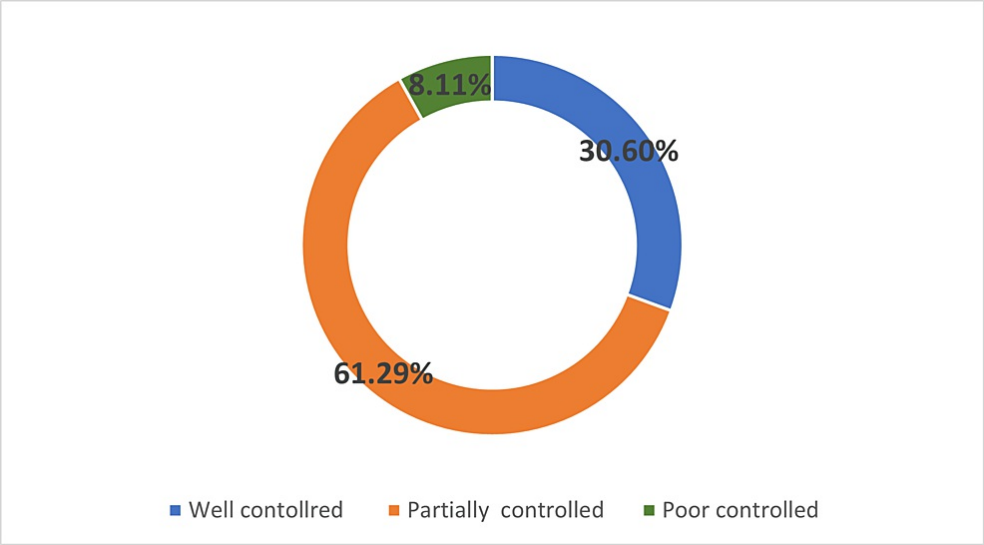
Asthma control among study children based on asthma control test

Figure 2 shows asthma control among participating children based on the asthma control test. Of the cohort, 30.60% had well-controlled asthma, whereas most of the children (61.29%) had partially controlled asthma.

Table 4 shows the risk factors of ER visits among participating children. Among all included factors, 81.1% of children did not regularly follow-up with a pediatrician and had visited the ER once or more compared to 54.9% of others who had followed up, with a recorded statistical significance (P=0.001). Also, 73.9% of children whose physician did not check the proper usage of their nebulizer or spacer visited the ER once or more versus 52.1% of others (P=0.022). Visiting the ER was reported among 28% of children who did not take a short-acting beta-agonist compared to 85% of those who needed more than one bottle per month (P=0.001). We found a significant relationship between controlled asthma and visiting the ER, in which well-controlled asthma patients visited the ER less frequently than those with partially or poorly controlled asthma (P=0.001). Likewise, 55.6% of the children who never had exercise-induced asthma exacerbation had visited the ER compared to those who had experienced exercise-induced asthma exacerbation (P=0.001).

**Table 4:** Risk factors of emergency department visit among asthmatic children P: Pearson X2 test ^Exact probability test *P<0.05 (significant)

Factors	Average visit to emergency department (yearly)										P-value
	Never		1-2 times		3-4 times		5-6 times		>6 times		
	No	%	No	%	No	%	No	%	No	%	
Age in years											.158
1-5	14	46.7%	5	16.7%	6	20.0%	2	6.7%	3	10.0%	
6-11	10	25.6%	14	35.9%	3	7.7%	5	12.8%	7	17.9%	
12-17	18	32.7%	19	34.5%	6	10.9%	9	16.4%	3	5.5%	
Gender											.601
Male	20	35.1%	15	26.3%	8	14.0%	6	10.5%	8	14.0%	
Female	22	32.8%	23	34.3%	7	10.4%	10	14.9%	5	7.5%	
On regular follow-up with pediatric clinic											.001*
No	10	18.9%	14	26.4%	8	15.1%	12	22.6%	9	17.0%	
Yes	32	45.1%	24	33.8%	7	9.9%	4	5.6%	4	5.6%	
Does your physician check the proper usage of nebulizer or spacer?											.022*
No	21	26.3%	25	31.3%	13	16.3%	14	17.5%	7	8.8%	
Yes	21	47.7%	13	29.5%	2	4.5%	2	4.5%	6	13.6%	
Usage of short-acting beta-agonist (monthly)											.001*^
Never	18	72.0%	5	20.0%	0	0.0%	0	0.0%	2	8.0%	
1-2 times	15	35.7%	14	33.3%	6	14.3%	6	14.3%	1	2.4%	
3-4 times	6	24.0%	7	28.0%	5	20.0%	3	12.0%	4	16.0%	
5-6 times	0	0.0%	7	58.3%	1	8.3%	3	25.0%	1	8.3%	
More than one bottle	3	15.0%	5	25.0%	3	15.0%	4	20.0%	5	25.0%	
Family history of asthma											.817
Yes	23	31.5%	24	32.9%	8	11.0%	11	15.1%	7	9.6%	
No	19	37.3%	14	27.5%	7	13.7%	5	9.8%	6	11.8%	
Home smoker											.155
Yes	10	23.8%	17	40.5%	6	14.3%	3	7.1%	6	14.3%	
No	32	39.0%	21	25.6%	9	11.0%	13	15.9%	7	8.5%	
Is there a pet in the house?											.084
Yes	9	29.0%	10	32.3%	5	16.1%	7	22.6%	0	0.0%	
No	33	35.5%	28	30.1%	10	10.8%	9	9.7%	13	14.0%	
Is your child exposed to triggers frequently?											.076
Yes	13	27.1%	17	35.4%	6	12.5%	10	20.8%	2	4.2%	
No	29	38.2%	21	27.6%	9	11.8%	6	7.9%	11	14.5%	
Average asthmatic exacerbation (yearly)											.001*^
Never	25	92.6%	2	7.4%	0	0.0%	0	0.0%	0	0.0%	
1-2 times	14	34.1%	21	51.2%	2	4.9%	3	7.3%	1	2.4%	
3-4 times	2	7.4%	13	48.1%	10	37.0%	0	0.0%	2	7.4%	
5-6 times	0	0.0%	0	0.0%	1	9.1%	8	72.7%	2	18.2%	
>6 times	1	5.6%	2	11.1%	2	11.1%	5	27.8%	8	44.4%	
Average exercise induces asthma exacerbation (yearly)											.001*^
Never	28	44.4%	21	33.3%	5	7.9%	7	11.1%	2	3.2%	
1-2 times	13	39.4%	11	33.3%	3	9.1%	3	9.1%	3	9.1%	
3-4 times	1	6.3%	6	37.5%	6	37.5%	2	12.5%	1	6.3%	
5-6 times	0	0.0%	0	0.0%	1	25.0%	1	25.0%	2	50.0%	
>6 times	0	0.0%	0	0.0%	0	0.0%	3	37.5%	5	62.5%	
Asthma control level											.001*^
Poorly controlled	0	0	0	0	5	50%	2	20%	3	30%	
Partially controlled	10	13.15%	7	9.2%	37	48.6%	15	19.7%	7	9.2%	
Well-controlled	8	21%	12	31.5%	13	34.2%	5	13.15%	0	0	

Table 5 shows risk factors of hospitalizations among participating children. A total of 47.2% of asthmatic children not regularly following up with pediatric clinics were hospitalized versus 21.1% of the others (P=0.014). Twenty percent of children never used short-acting beta-agonists, whereas 45% needed more than one bottle (P=0.005). Also, 41.5% of children with a smoker at home were hospitalized compared to 28% who did not (P=0.024). A total of 34.5% of children with a pet in the house were hospitalized versus 31.2% of those who did not (P=0.041). Additionally, none of the children who had never had asthmatic exacerbation were hospitalized versus 21.8% of those who had five to six exacerbations (P=0.001). Also, 23.8% of children who never had exercise-induced asthma exacerbation needed hospitalization compared to 75% of others who had five to six times (P=0.001).

**Table 5:** Risk factors of hospitalizations among asthmatic children P: Pearson X2 test ^Exact probability test *P<0.05 (significant)

Factors	Average hospitalization (yearly)										P-value
	Never		1-2 times		3-4 times		5-6 times		>6 times		
	No	%	No	%	No	%	No	%	No	%	
Age in years											.392
1-5	20	66.7%	4	13.3%	2	6.7%	0	0.0%	4	13.3%	
6-11	28	71.8%	5	12.8%	4	10.3%	1	2.6%	1	2.6%	
12-17	36	65.5%	12	21.8%	5	9.1%	1	1.8%	1	1.8%	
Gender											.751
Male	40	70.2%	9	15.8%	5	8.8%	0	0.0%	3	5.3%	
Female	44	65.7%	12	17.9%	6	9.0%	2	3.0%	3	4.5%	
On regular follow-up with pediatric clinic											.014*
No	28	52.8%	11	20.8%	7	13.2%	2	3.8%	5	9.4%	
Yes	56	78.9%	10	14.1%	4	5.6%	0	0.0%	1	1.4%	
Dose your physician check the proper usage of nebulizer or spacer?											.049*
No	48	60.0%	19	23.8%	7	8.8%	2	2.5%	4	5.0%	
Yes	36	81.8%	2	4.5%	4	9.1%	0	0.0%	2	4.5%	
Usage of short-acting beta-agonist (monthly)											.005*
Never	20	80.0%	2	8.0%	2	8.0%	0	0.0%	1	4.0%	
1-2 times	28	66.7%	10	23.8%	4	9.5%	0	0.0%	0	0.0%	
3-4 times	16	64.0%	5	20.0%	4	16.0%	0	0.0%	0	0.0%	
5-6 times	9	75.0%	2	16.7%	0	0.0%	1	8.3%	0	0.0%	
More than one bottle	11	55.0%	2	10.0%	1	5.0%	1	5.0%	5	25.0%	
Family history of asthma											.441
Yes	48	65.8%	14	19.2%	7	9.6%	0	0.0%	4	5.5%	
No	36	70.6%	7	13.7%	4	7.8%	2	3.9%	2	3.9%	
Home smoker											.024*
Yes	25	59.5%	10	23.8%	2	4.8%	0	0.0%	5	11.9%	
No	59	72.0%	11	13.4%	9	11.0%	2	2.4%	1	1.2%	
Is there a pet in the house											.041*
Yes	20	64.5%	10	32.3%	1	3.2%	0	0.0%	0	0.0%	
No	64	68.8%	11	11.8%	10	10.8%	2	2.2%	6	6.5%	
Dose your child expose to triggers frequently?											.306
Yes	32	66.7%	10	20.8%	5	10.4%	1	2.1%	0	0.0%	
No	52	68.4%	11	14.5%	6	7.9%	1	1.3%	6	7.9%	
Average asthmatic exacerbation (yearly)											.001*
Never	27	100.0%	0	0.0%	0	0.0%	0	0.0%	0	0.0%	
1-2 times	34	82.9%	4	9.8%	2	4.9%	1	2.4%	0	0.0%	
3-4 times	13	48.1%	11	40.7%	3	11.1%	0	0.0%	0	0.0%	
5-6 times	2	18.2%	4	36.4%	5	45.5%	0	0.0%	0	0.0%	
>6 times	8	44.4%	2	11.1%	1	5.6%	1	5.6%	6	33.3%	
Average exercise induces asthma exacerbation (yearly)											.001*
Never	48	76.2%	7	11.1%	4	6.3%	1	1.6%	3	4.8%	
1-2 times	19	57.6%	11	33.3%	3	9.1%	0	0.0%	0	0.0%	
3-4 times	10	62.5%	3	18.8%	2	12.5%	0	0.0%	1	6.3%	
5-6 times	1	25.0%	0	0.0%	2	50.0%	1	25.0%	0	0.0%	
>6 times	6	75.0%	0	0.0%	0	0.0%	0	0.0%	2	25.0%	
Asthma control level											.525
Poorly controlled	1	10%	8	80%	1	10%	0	0	0	0	
Partially controlled	30	39.4%	27	35.5%	6	7.8%	12	15.7%	1	1.3%	
Well-controlled	15	39.4%	10	26.3%	10	26..3%	3	7.8%	0	0	

## Discussion

Asthma is a chronic respiratory disease affecting children and adults with an estimated worldwide prevalence of 10%. In 2019, it affected over 600 million people. The United Kingdom has one of the highest reported prevalences in Western Europe, leading to significant morbidity and mortality. Asthma accounts for over 1,000 deaths and 60,000 emergency hospital admissions annually [2]. This results in a considerable health and economic burden of 200,000 bed-days per year and costs the United Kingdom National Health Service £1.1 billion annually. Despite the implementation of national clinical guidelines and recommendations, there has been little reduction in asthma attacks across all age groups [15-17]. In the current study, we aimed to assess risk factors of ER visits and hospital admission among asthmatic children. The study showed that most of the children aged six years or more were girls. Also, one-third of the children had nasal allergies, and one-fourth of them complained of eczema, whereas less than half of them had no other chronic health problem. Simms-Williams et al. reported that 11.4% of asthmatic children had allergies, and 6.8% complained of eczema [18].

As for asthma clinical profiles and severity, we found that most of the asthmatic children and their parents did not know about the severity, and nearly one-tenth had a severity score of four or five. More than two-thirds of the asthmatic children were exposed to triggers frequently. About one-fourth experienced five or more asthmatic exacerbations per year and visited the ER five times or more per year. A low percentage of asthmatic children were hospitalized five times or more per year. ICU admission due to asthma was infrequent, and few children needed mechanical ventilation. Almost half were investigated for aeroallergens, which revealed that most of them were sensitive to dust. Regarding risk factors, more than half of the children had a family history of asthma, one-third had smokers at home, and one-fourth of them had a pet in the house. Similar low ER visits and hospital admission rates were reported by Simms-Williams et al. as 3.2% of children and 1.2% of adolescents experienced at least one incident asthma-related hospital admission during the three-year follow-up period [18]. Also, Bloom et al. documented that more than 60% of the participating children had mild asthma, and most of the young asthmatic patients had not experienced an exacerbation during follow-up [19]. This was confirmed by Hasegawa et al. because after being admitted for asthma-related issues, 14.5% of patients were readmitted within 30 days due to a wide range of diagnoses [20]. Other studies showed that older adults with asthma have more severe symptoms, worse control and response to medication, and reduced lung function compared to younger people [21-24].

With regard to risk factors associated with ER visits and hospitalization, the current study showed that those who did not follow-up with pediatricians were using short-acting beta-agonists at a high rate and had a physician who did not regularly check the proper usage of nebulizers or spacers were associated with higher rates of ER visits and hospitalization. Meanwhile, experiencing more frequent asthmatic exacerbation and exercise-induced asthma exacerbation were associated with a higher frequency of ED visits and hospitalization. Also, having a home smoker and a pet in the house was associated with a higher hospitalization rate. Similar findings were reported by Aggarwal et al., who found that exposure to environmental tobacco smoke, pet dander, previous asthma hospitalizations, poor asthma control, higher severity symptoms at ER presentations, and a warmer season at admission were significant factors associated with future hospitalization among children with asthma [25]. Simms-Williams et al. found that being a girl is an important risk factor for asthma-related hospital admissions, which was also reported by other two studies [18,26,27].

One of the limitations of this study is generalizability. It should be carried out among a larger sample size and include participants from different regions in Saudi Arabia. Furthermore, when it comes to asthma control, qualitative research may yield more accurate results than semi-structured questionnaires. Because questionnaires were used to gather the data, recollection bias could have affected the results. In practical practice, the ACT should be used in conjunction with a clinician's assessment to appropriately reflect patients' asthma control levels. These findings show that educational initiatives and increased awareness among the general public in general, and caregivers in particular, are needed to enhance their knowledge and, as a result, the asthma control status of their children.

## Conclusions

The current study revealed that asthma among children was mainly of low severity with most having a controlled case of asthma. This finding means that asthmatic children had infrequent ER visits. A higher rate of exacerbations, exposure to smoke, and home pets were the most significant factors associated with higher ER visits and hospitalizations. Periodic screening and treatment of asthma risk factors could lead to better asthma outcomes and fewer unnecessary hospital admissions. Additionally, tracking high-risk children for admission and readmission is an important measure that can control poor outcomes. As such, it should be a key consideration in monitoring and supporting individuals with asthma.
